# Review of the Welsh mammographic screening programme for women who underwent mantle irradiation for Hodgkin lymphoma

**DOI:** 10.1186/bcr3298

**Published:** 2012-11-09

**Authors:** GM Edwards, E Gallop-Evans, C Jordan, G Stevens, K Gower Thomas

**Affiliations:** 1 Breast Test Wales, Cardiff, UK; 2 Velindre Cancer Centre, Cardiff, UK; 3 University of Wales Medical School, Cardiff, UK

## Introduction

There is evidence that women who received supra-diaphragmatic radiotherapy (sRT) for Hodgkin lymphoma at a young age are at increased risk of developing breast cancer. This study reports the implementation and results of the breast screening programme for such women in South Wales, which offers screening to women eight or more years following treatment.

## Methods

Women were identified from the hospital Hodgkin Lymphoma and the Breast Test Wales screening databases. Those treated with sRT below the age of 36 years were included in the review (sRT range 9 to 34 years.) Breast cancer and cause of death developing in the cohort were recorded.

## Results

Ninety-four eligible women were identified who underwent sRT between 1977 and 2004 in whom seven breast cancers developed. Three were found on self-examination, two through the Hodgkin screening programme, one through NHS screening and one had incomplete data. Sixteen women died, three from breast cancer. The mean induction period was 21.4 years between radiation and breast cancer presentation.

## Conclusion

This review confirms an increased incidence of breast cancer in these irradiated women many years following the therapy. The need for a nationwide screening programme is confirmed although the age at which to offer screening could be reviewed.

